# Maternal Psychological Distress Before and After Childbirth and Neurodevelopmental Delay in Toddlers

**DOI:** 10.1001/jamanetworkopen.2025.40907

**Published:** 2025-10-31

**Authors:** Kenta Matsumura, Tomomi Tanaka, Masami Kuroda, Akiko Tsuchida, Takehiro Hatakeyama, Haruka Kasamatsu, Hidekuni Inadera

**Affiliations:** 1 Graduate School of Health Sciences, Aomori University of Health and Welfare, Aomori, Japan; 2 Department of Public Health, Faculty of Medicine, University of Toyama, Toyama, Japan; 3 Toyama Regional Center for Japan Environment and Children's Study, University of Toyama, Toyama, Japan; 4 Department of Pediatrics, Faculty of Medicine, University of Toyama, Toyama, Japan; 5 now with Shinseikai Toyama Hospital, Imizu, Japan; 6 Research Center for Regional Co-creation Basis, Shibaura Institute of Technology Research Laboratories, Shibaura Institute of Technology, Tokyo, Japan

## Abstract

**Importance:**

Maternal psychological distress is a risk factor for neurodevelopmental delay in the offspring, but no previous studies have examined this while considering the bidirectional relationship between psychological distress and child development over time.

**Objective:**

To estimate the risk of neurodevelopmental delay in toddlers following maternal psychological distress during mid- to late pregnancy and at 1 year post partum.

**Design, Setting, and Participants:**

This birth cohort study, the Japan Environment and Children’s Study (JECS), is an ongoing nationwide longitudinal cohort. Recruitment was conducted through 15 regional centers across Japan from January 2011 to March 2014. Participants were 82 418 mother-child pairs enrolled in the JECS. Data were analyzed from July 2024 to July 2025.

**Exposures:**

Psychological distress, defined as a Kessler Psychological Distress Scale (K6) score of 5 or greater.

**Main Outcomes and Measures:**

Neurodevelopmental delay, defined as a score less than the cutoff in any of 5 developmental areas identified at any of 4 measurement times (ages 1.5, 2.0, 2.5, and 3.0 years), using the Ages and Stages Questionnaire, Third Edition. A marginal structural modeling approach was used.

**Results:**

Among 82 418 mother-child pairs, the mothers’ mean (SD) age was 31.1 (5.0) years, and 40 125 children (48.7%) were female. Compared with having no psychological distress at either time point, the adjusted odds ratio was higher when psychological distress was present at 1 year post partum only (aOR, 1.25; 95% CI, 1.18-1.33) than when it was present during mid- to late pregnancy only (aOR, 1.08; 95% CI, 1.03-1.13). The aOR for psychological distress at both time points was 1.38 (95% CI, 1.30-1.46). No salient multiplicative interaction between the 2 exposure periods was observed (aOR, 1.02; 95% CI, 0.93-1.11).

**Conclusions and Relevance:**

In this cohort study of 82 418 mother-child pairs, maternal psychological distress at 1 year post partum had an estimated higher risk of neurodevelopmental delay in toddlers than psychological distress during pregnancy. These findings suggest the importance of maintaining maternal mental health from pregnancy through 1 year post partum.

## Key Points

**Question:**

Is the timing of maternal psychological distress differentially associated with the risk of neurodevelopmental delay in toddlers?

**Findings:**

In this birth cohort study of 82 418 mother-child pairs, maternal psychological distress at 1 year post partum had an estimated higher risk of neurodevelopmental delay in toddlers than psychological distress during mid- to late pregnancy, with no salient multiplicative interaction between time points observed.

**Meaning:**

These findings suggest the importance of maintaining maternal mental health from pregnancy through 1 year post partum.

## Teaser

This cohort study evaluates whether the timing of maternal psychological distress—ie, during mid- to late pregnancy or during the postpartum period—is associated with the risk of neurodevelopment delay in offspring at ages 1.5 to 3.0 years.

## Introduction

Compared with other mammals, humans are physiologically premature at birth.^1^ Although human infants need to be closely monitored by their caregivers for an extended developmental period, this facilitates considerable plasticity in development. Indeed, the rapid brain development that starts in the fetal period continues until approximately 2 years of age,^2,3^ and this secondary (late) stage of development, which builds on the first (early) stage, enables flexibility in the acquisition of knowledge, thoughts, behaviors, language, and social skills.

During these crucial developmental periods, maternal mental health problems can hinder children’s development. Maternal psychological distress—including depression, anxiety, and/or stress—during the prenatal and postpartum periods can negatively affect children’s social, emotional, linguistic, and cognitive development.^4,5,6,7,8,9^ This association can involve multiple pathways, including disrupted parenting behavior and mother-infant interaction,^10,11,12,13^ reduced duration of breastfeeding,^14,15^ and biological mechanisms, such as cortisol overproduction, leading to fetal stunting and preterm birth.^7,16^ Meanwhile, child-related factors, such as low birth weight, preterm birth,^17,18^ and delayed child development,^19^ can increase maternal psychological distress, indicating a complex bidirectional relationship between mother and child. Furthermore, distress during pregnancy can also lead to postpartum distress,^20,21,22^ and early developmental delays may persist over time.^23^ However, despite these complex time-varying and bidirectional causal relationships, few studies have examined whether prenatal or postpartum maternal psychological distress has a greater impact on neurodevelopmental delay in toddlers. This gap contrasts with the numerous studies that have assessed maternal psychological distress either before childbirth or after,^24,25,26,27^ or both,^28,29,30,31^ often without a causal framework. Understanding the timing of these effects might have important implications for the design of interventions^5^ to prevent developmental delays in children.

In this study, we used data from the Japan Environment and Children’s Study (JECS), an ongoing nationwide birth cohort study, to examine this complex bidirectional relationship between maternal psychological distress and child neurodevelopment. Repeated measures of maternal psychological distress (during mid- to late pregnancy and at 1 year post partum) and child neurodevelopment (from age 6 months to 3 years) were analyzed using a marginal structural model with inverse probability of treatment weighting.^32,33^ Our objective was to estimate the effect of psychological distress at each time point on neurodevelopmental delay in toddlers, including potential interactions between time points.

## Methods

### Study Design

The JECS is a government-funded, nationwide, birth cohort study that recruited participants from 15 regional centers across Japan from January 2011 to March 2014, registering 103 057 pregnancies. Expectant mothers residing outside the study area were excluded. The study investigates the association of environmental factors with children’s health and development. Detailed descriptions of the study can be found elsewhere.^34,35^ All procedures involving human participants for the JECS protocol were approved by the Institutional Review Board on Epidemiological Studies of the Japanese Ministry of the Environment and the ethics committees of all participating institutions. Written informed consent was obtained from all participants. The protocol of the present study was also approved by the Ethics Committee of University of Toyama. The study followed the Strengthening the Reporting of Observational Studies in Epidemiology (STROBE) reporting guideline for cohort studies.

### Study Data

**Figure 1.**
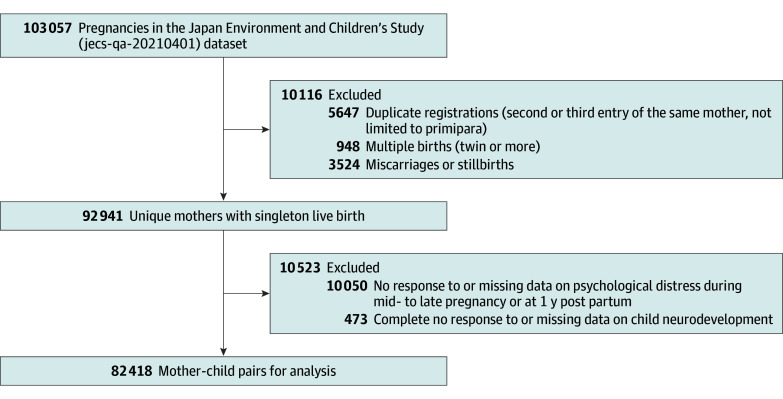
Participant Flow Diagram The jecs-qa-20210401 dataset was first released in October 2019 and completed in March 2022.

This study used the jecs-qa-20210401 dataset, which was first released in October 2019 and completed in March 2022. Data on 82 418 mother-child pairs (Figure 1) were analyzed from July 2024 to July 2025.

### Measurements

The mothers completed a self-administered questionnaire on 10 occasions—during early and mid- to late pregnancy, at 0 and 1 month post partum, and at 0.5, 1.0, 1.5, 2.0, 2.5, and 3.0 years post partum—to collect information on demographic and socioeconomic status, medical and obstetric history, the physical and mental health of both mother and child, lifestyle, and other aspects. Up to 1 month post partum, the questionnaires were distributed to the participants by JECS staff and were then collected at the cooperating health care practitioners when the participants visited for health checkups. Questionnaires were returned by postal mail if they could not be collected at that time. From 0.5-years post partum, the questionnaires were distributed and collected by postal mail.

#### Exposures

Psychological distress during mid- to late pregnancy and at 1 year post partum was measured using the Kessler 6 (K6) questionnaire,^36^ a 6-item self-administered questionnaire developed and validated to detect general psychological distress, with total scores ranging from 0 to 24. The Japanese version of the K6 has also been validated,^37^ an optimal cut-off score between 4 and 5, defined as the score that maximizes the sum of sensitivity and specificity, has been established, and good discriminative ability for mood and anxiety disorders has been demonstrated, with 100% sensitivity and 69% specificity.^38^ We defined psychological distress as a K6 score of 5 or greater.

#### Outcomes

Children’s neurodevelopment during ages 1.5 to 3.0 years was assessed every 6 months, using the Ages and Stages Questionnaire, Third Edition (ASQ-3),^23^ an age-specific, structured, parent-completed child-monitoring system. The ASQ-3 is a set of well-validated questionnaires recommended by the United Nations Children’s Fund to verify whether children have typical neurological development^39^ and covers 5 areas of development: communication, gross motor, fine motor, problem solving, and personal-social skills. Each domain has 6 items, which are rated from 0 to 10, for a total domain score of 0 to 60 (higher is better). The Japanese version of the ASQ-3 has also been validated, cutoff values have been established, and moderate to high sensitivity (75.3%-100.0%) and specificity (66.6%-100.0%) have been demonstrated for neurodevelopmental delay.^40^ The primary outcome of this study is neurodevelopmental delay, defined as a score below the cutoff in any of the 5 developmental areas at any of the 4 measurement time points (ie, ages 1.5, 2.0, 2.5, and 3.0 years). The secondary outcomes were 20 individual delays, one detected in each developmental area and time point.

#### Covariates

We selected potential baseline confounders, defined as variables before and/or during pregnancy with a theoretical impact on both exposure and outcome, as well as basic anthropometric and socioeconomic variables. In addition, we selected covariates for childbirth to 1 year post partum, defined as variables that appeared after baseline or time-dependent variables that can be affected by previous variables. All variables were categorized according to standard clinical practice, common practice in Japan, and/or previous studies.^41^

### Statistical Analysis

The participants’ characteristics are summarized using frequencies and percentages. Differences between pregnant women who were included and those who were excluded were examined using Cramer *V*, calculated from the χ^2^ value.

**Figure 2.**
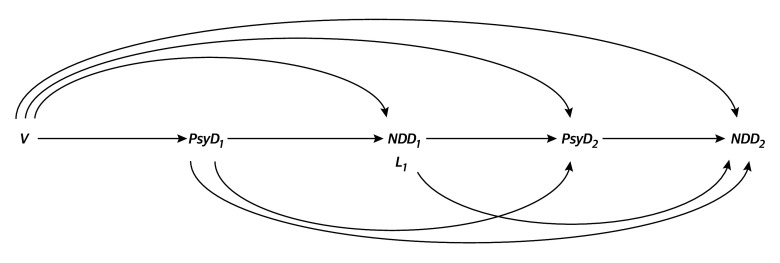
Directed Acyclic Graph Diagram illustrating the associations of time-varying psychological distress (PsyD) during mid- to late pregnancy (PsyD_1_) and at 1 year post partum (PsyD_2_) with neurodevelopment delay (NDD) during age 0.5 to 1.0 years (NDD_1_) as a time-varying covariate that is simultaneously a confounder and intermediate variable and NDD at age 1.5 to 3.0 years (NDD_2_). PsyD was set at 1 if the mother had psychological distress and 0 otherwise. NDD was set at 1 if the child had neurodevelopmental delay and 0 otherwise. *V* represents baseline confounders; L_1_ represents time-varying covariates, similar to NDD_1_, which appeared after PsyD_1_ and are simultaneously confounders and intermediate variables.

Marginal structural models in combination with inverse probability of treatment weighting were used to estimate the association of psychological distress during mid- to late pregnancy and at 1 year post partum as well as their interaction with the risk of neurodevelopmental delay.^32,33,42,43^ These were used to adequately disentangle complex time-varying and bidirectional associations among variables.^44,45^ Figure 2 shows a directed acyclic graph of this study. The marginal structural model took the following form:

logit Pr[NDD_2_^PsyD1,PsyD2^ = 1] = β_0_ + (β_1_ × PsyD_1_) + (β_2_ × PsyD_2_) + (β_3_ × PsyD_1_ × PsyD_2_),

where NDD_2_ indicates neurodevelopment delay from ages 1.5 to 3.0 years; PsyD_1_, maternal psychological distress during mid- to late pregnancy; and PsyD_2_, maternal psychological distress at 1 year post partum. NDD_2_^PsyD1,PsyD2^ is the counterfactual outcome of neurodevelopmental delay (1 if the child had NDD; otherwise 0) if the mothers had experienced psychological distress, contrary to the fact that could set PsyD_1_ and PsyD_2_ to 1 or 0, respectively (1 if the mother had psychological distress, otherwise 0). Here, psychological distress is estimated during mid- to late pregnancy only, at 1 year post partum only, and at both time points, expressed as counterfactual odds ratios (ORs) exp(β_1_), exp(β_2_)_,_ and exp(β_1_ + β_2_ + β_3_), respectively. Exp(β_3_) represents the multiplicative interaction between psychological distress at both time points. References were set to the absence of psychological distress at both time points, ie, PsyD_1_ = PsyD_2_ = 0. Robust variance estimates were used to calculate 95% CIs.

When fitting the marginal structural models, we used stabilized inverse probability weighting (SW), defined as:
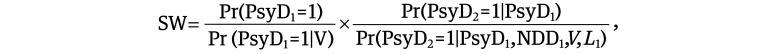
where *V* represents the baseline potential confounders (mother’s age during pregnancy; prepregnancy body mass index; parity; highest education level^46^; annual household income; marital status; physical activity^47,48^; alcohol intake; smoking history; passive smoking; employment status; history of major psychiatric disorders, such as depression, anxiety, and schizophrenia; autistic trait assessed using the Autism-Spectrum Quotient^49^; folic acid intake^50^ and energy-adjusted omega-3 polyunsaturated fatty acid intake,^51,52^ both assessed using the Food Frequency Questionnaire^53^; emotional social support^54^; negative feelings upon learning of pregnancy; intimate partner violence^55^; living with the mother’s parent[s]; living with the partner’s parent[s]; pet ownership^56^; stressful event; and area of residence [1 of 15 regional centers where participants were recruited]), and *L_1_* represents post-childbirth time-varying covariates that are simultaneously confounders and intermediate variables (cesarean delivery; preterm birth; low birth weight; child sex; 5-minute Apgar score^57^; major congenital anomaly^58^; feeding method^59^; nursery attendance; marital status; employment status; child living with the mother or father; child living with siblings; child living with the mother’s parent[s]; child living with the father’s parent[s]; pet ownership; and stressful event), as shown in Figure 2. NDD_1_, also a confounder and an intermediate variable, represents neurodevelopmental delay during ages 0.5 to 1.0 years.

We created 4 types of SWs and referred to the corresponding models as follows: (1) fully adjusted model, estimating adjusted ORs (aORs) using SW incorporating all covariates; (2) partially adjusted model, estimating partially adjusted ORs using SW based on selected key variables from model 1; (3) crude model, estimating crude ORs using SW excluding covariates (based only PsyD_1_, PsyD_2_, and NDD); and (4) unweighted model, estimating reference ORs using SW fixed at 1. To prevent estimation instability caused by outliers, SW was truncated at the 1st and 99th percentiles.

For the primary outcome analysis, the marginal structural model was fitted using neurodevelopmental delay during ages 1.5 to 3.0 years as the outcome (NDD_2_) and delay during ages 0.5 to 1.0 years as a confounder and intermediate variable (NDD_1_). For the secondary outcome analysis, a separate 20 sets of marginal structural models were fitted by combining 20 cases of each neurodevelopmental delay (ie, delay in each developmental area and at each time point) as NDD_2_ and delay during ages 0.5 to 1.0 years in the corresponding domains as NDD_1_. Although 20 repeated analyses were performed, no correction for multiple comparisons was applied because of the secondary analysis.

Missing data were treated using multiple imputation. The missing data rate in the main analysis was approximately 4% or less for all variables, excluding 5.0% for 5-minute Apgar score, 5.0% for stressful event (second measurement), and 6.7% for income. The mean effective response rates at children’s ages of 1.5, 2.0, 2.5, and 3.0 years were 83.1%, 86.8%, 84.7%, and 86.5%, respectively. Ten imputed datasets were created using chained equations while including auxiliary variables related to missing variables to prevent violation of the assumption of missing at random.^60^ The results were combined using standard rules.^61^

To assess the robustness of the findings, we performed several sensitivity analyses: redefining psychological distress as a K6 score of 13 or greater (65% sensitivity and 97% specificity)^38^ and using a continuous severity score of neurodevelopmental delay defined as 0 (0 domains below cutoff), 1 (1 domain), 2 (2-4 domains), and 3 (≥5 domains), based on scores across 5 developmental domains and 4 time points, instead of the binary classification used in the main analysis. To evaluate unmeasured confounding, we calculated the E-value.^62^ We also examined the SW range, distribution of propensity scores, and covariate balance.

All data were analyzed using SAS version 9.4 (SAS Institute) and R version 4.5.1 (R Foundation for Statistical Computing). We provide SEMs and 95% CIs with our results, but we did not set a prespecified level of statistical significance.

## Results

In total, 82 418 mother-child pairs were analyzed; the mothers’ mean (SD) age was 31.1 (5.0) years, and their mean prepregnancy body mass index (BMI; calculated as weight in kilograms divided by height in meters squared) was 21.2 (3.2). A total of 40 125 children (48.7%) were female. Table 1 shows the participants’ characteristics according to psychological distress. A total of 23 365 mothers (28.3%) reported psychological distress during mid- to late pregnancy only, 18 201 (22.1%) at 1 year post partum only, and 10 496 (12.7%) at both. Neurodevelopmental delay was observed in 23 007 children (27.9%). Compared with the analyzed mothers, those who were excluded tended to smoke (Cramer *V* = 0.117), be younger (Cramer *V* = 0.105), be less educated (Cramer *V* = 0.100), be a passive smoker (Cramer *V* = 0.096), and have lower income (Cramer *V* = 0.055).

**Table 1.** Characteristics of Participants According to Maternal Psychological Distress at Different Time Points

Characteristic	Psychological distress, No. (%)			
	During mid- to late pregnancy only		At 1 y post partum only	
	No (n = 59 053)	Yes (n = 23 365)	No (n = 64 217)	Yes (n = 18 201)
Measured during pregnancy				
Maternal age, y				
<25	4694 (8.0)	3155 (13.5)	5708 (8.9)	2141 (11.8)
25 to <30	16 237 (27.5)	7037 (30.1)	17 877 (27.8)	5397 (29.7)
30 to <35	21 631 (36.6)	7821 (33.5)	23 195 (36.1)	6257 (34.4)
≥35	16 491 (27.9)	5352 (22.9)	17 437 (27.2)	4406 (24.2)
Prepregnancy BMI				
<18.5	9496 (16.1)	3867 (16.6)	10 365 (16.1)	2998 (16.5)
18.5 to <25	43 895 (74.3)	16 858 (72.2)	47 498 (74.0)	13 255 (72.8)
≥25	5662 (9.6)	2641 (11.3)	6355 (9.9)	1948 (10.7)
Parity				
0	25 919 (43.9)	10 864 (46.5)	28 635 (44.6)	8148 (44.8)
1	22 028 (37.3)	8106 (34.7)	23 317 (36.3)	6817 (37.5)
≥2	11 106 (18.8)	4395 (18.8)	12 266 (19.1)	3235 (17.8)
Highest education level, y				
≤12	19 191 (32.5)	9418 (40.3)	21 678 (33.8)	6931 (38.1)
12 to <16	25 823 (43.7)	9350 (40.0)	27 916 (43.5)	7257 (39.9)
≥16	14 039 (23.8)	4597 (19.7)	14 623 (22.8)	4013 (22.1)
Annual household income, millions of ¥^a^				
<4	22 021 (37.3)	10 701 (45.8)	24 660 (38.4)	8062 (44.3)
4 to <6	20 083 (34.0)	7301 (31.3)	21 503 (33.5)	5881 (32.3)
≥6	16 950 (28.7)	5363 (23.0)	18 054 (28.1)	4259 (23.4)
Marital status				
Married	57 043 (96.6)	21 812 (93.4)	61 691 (96.1)	17 164 (94.3)
Unmarried	2010 (3.4)	1553 (6.7)	2526 (3.9)	1037 (5.7)
Physical activity				
No	13 542 (22.9)	5484 (23.5)	15 137 (23.6)	3888 (21.4)
Yes	45 511 (77.1)	17 882 (76.5)	49 080 (76.4)	14 313 (78.6)
Alcohol intake				
Never	20 149 (34.1)	7547 (32.3)	21 815 (34.0)	5881 (32.3)
Former	37 376 (63.3)	15 101 (64.6)	40 698 (63.4)	11 779 (64.7)
Current	1528 (2.6)	717 (3.1)	1704 (2.7)	541 (3.0)
Smoking history				
Never	36 255 (61.4)	12 496 (53.5)	38 629 (60.2)	10 122 (55.6)
Former	20 898 (35.4)	9542 (40.8)	23 291 (36.3)	7149 (39.3)
Current	1901 (3.2)	1328 (5.7)	2298 (3.6)	931 (5.1)
Passive smoking				
Almost never	38 893 (65.9)	13 169 (56.4)	41 469 (64.6)	10 592 (58.2)
Once a week	6873 (11.6)	3013 (12.9)	7505 (11.7)	2381 (13.1)
2-3 Times a week	4353 (7.4)	2269 (9.7)	4954 (7.7)	1668 (9.2)
4-6 Times a week	2672 (4.5)	1302 (5.6)	2948 (4.6)	1026 (5.6)
Everyday	6262 (10.6)	3613 (15.5)	7340 (11.4)	2535 (13.9)
Employed				
No	26 207 (44.4)	11 501 (49.2)	28 581 (44.5)	9127 (50.2)
Yes	32 846 (55.6)	11 864 (50.8)	35 636 (55.5)	9074 (49.9)
History of major psychiatric disorders				
No	55 734 (94.4)	20 324 (87.0)	60 456 (94.1)	15 602 (85.7)
Yes	3319 (5.6)	3041 (13.0)	3761 (5.9)	2599 (14.3)
Autistic trait				
No	57 944 (98.1)	22 307 (95.5)	62 932 (98.0)	17 319 (95.2)
Yes	1109 (1.9)	1058 (4.5)	1285 (2.0)	882 (4.8)
Folic acid intake, quintile^b^				
First (≤152 mg/d)	11 683 (19.8)	4831 (20.7)	13 021 (20.3)	3493 (19.2)
Second (152-203 mg/d)	11 973 (20.3)	4534 (19.4)	13 058 (20.3)	3449 (19.0)
Third (203-257 mg/d)	12 103 (20.5)	4413 (18.9)	12 861 (20.0)	3656 (20.1)
Fourth (257-338 mg/d)	11 824 (20.0)	4596 (19.7)	12 666 (19.7)	3755 (20.6)
Fifth (>338 mg/d)	11 470 (19.4)	4990 (21.4)	12 611 (19.6)	3849 (21.2)
Energy-adjusted omega-3 PUFA intake, quintile^b^				
First (≤1.18 g/d)	11 328 (19.2)	5155 (22.1)	12 639 (19.7)	3844 (21.1)
Second (1.17-1.45 g/d)	11 628 (19.7)	4856 (20.8)	12 673 (19.7)	3812 (20.9)
Third (1.44-1.69 g/d)	11 956 (20.3)	4528 (19.4)	12 838 (20.0)	3646 (20.0)
Fourth (>1.68-2.01 g/d)	12 056 (20.4)	4429 (19.0)	13 085 (20.4)	3399 (18.7)
Fifth (>2.00 g/d)	12 086 (20.5)	4397 (18.8)	12 983 (20.2)	3500 (19.2)
Emotional social support				
Low	15 085 (25.5)	9169 (39.2)	17 098 (26.6)	7156 (39.3)
Mid-low	11 985 (20.3)	5118 (21.9)	13 138 (20.5)	3965 (21.8)
Mid-high	16 909 (28.6)	5106 (21.9)	17 932 (27.9)	4083 (22.4)
High	15 075 (25.5)	3972 (17.0)	16 049 (25.0)	2998 (16.5)
Negative feelings upon learning of pregnancy				
No	54 935 (93.0)	20 347 (87.1)	59 456 (92.6)	15 827 (87.0)
Yes	4118 (7.0)	3018 (12.9)	4761 (7.4)	2374 (13.0)
Intimate partner violence				
No	53 565 (90.7)	17 654 (75.6)	57 098 (88.9)	14 120 (77.6)
Yes	5489 (9.3)	5711 (24.4)	7119 (11.1)	4081 (22.4)
Living with mother’s parent(s)				
No	53 535 (90.7)	20 383 (87.2)	57 773 (90.0)	16 146 (88.7)
Yes	5518 (9.3)	2982 (12.8)	6444 (10.0)	2055 (11.3)
Living with partner’s parent(s)				
No	52 388 (88.7)	20 379 (87.2)	56 882 (88.6)	15 885 (87.3)
Yes	6665 (11.3)	2986 (12.8)	7336 (11.4)	2316 (12.7)
Pet ownership type				
None	48 937 (82.9)	18 493 (79.2)	52 760 (82.2)	14 670 (80.6)
Dog(s) only	6419 (10.9)	2929 (12.5)	7265 (11.3)	2083 (11.4)
Cat(s) only	2810 (4.8)	1454 (6.2)	3161 (4.9)	1103 (6.1)
Both	887 (1.5)	489 (2.1)	1031 (1.6)	345 (1.9)
Stressful event				
No	36 891 (62.5)	9658 (41.3)	38 719 (60.3)	7831 (43.0)
Yes	22 162 (37.5)	13 707 (58.7)	25 498 (39.7)	10 371 (57.0)
Area of residence				
A	4805 (8.1)	1756 (7.5)	5247 (8.2)	1314 (7.2)
B	4710 (8.0)	2264 (9.7)	5407 (8.4)	1567 (8.6)
C	7485 (12.7)	3581 (15.3)	8386 (13.1)	2680 (14.7)
D	3115 (5.3)	1576 (6.8)	3458 (5.4)	1233 (6.8)
E	3813 (6.5)	1637 (7.0)	4070 (6.3)	1380 (7.6)
F	4241 (7.2)	1619 (6.9)	4596 (7.2)	1264 (6.9)
G	3403 (5.8)	1247 (5.3)	3562 (5.6)	1088 (6.0)
H	3338 (5.7)	1229 (5.3)	3485 (5.4)	1082 (5.9)
I	2397 (4.1)	838 (3.6)	2559 (4.0)	676 (3.7)
J	4689 (7.9)	1694 (7.3)	5091 (7.9)	1292 (7.1)
K	3125 (5.3)	1075 (4.6)	3275 (5.1)	925 (5.1)
L	1792 (3.0)	728 (3.1)	1886 (2.9)	634 (3.5)
M	4192 (7.1)	1382 (5.9)	4462 (7.0)	1112 (6.1)
N	4643 (7.9)	1514 (6.5)	5120 (8.0)	1037 (5.7)
O	3305 (5.6)	1225 (5.2)	3613 (5.6)	917 (5.0)
Measured between delivery and 1 y post partum				
Cesarean section				
No	48 072 (81.4)	19 012 (81.4)	52 280 (81.4)	14 804 (81.3)
Yes	10 981 (18.6)	4353 (18.6)	11 937 (18.6)	3397 (18.7)
Preterm birth (<37 wk)				
No	56 607 (95.9)	22 200 (95.0)	61 448 (95.7)	17 359 (95.4)
Yes	2447 (4.1)	1165 (5.0)	2769 (4.3)	842 (4.6)
Low birth weight (<2500 g)				
No	54 550 (92.4)	21 371 (91.5)	59 187 (92.2)	16 734 (91.9)
Yes	4503 (7.6)	1994 (8.5)	5031 (7.8)	1467 (8.1)
Child sex				
Female	28 828 (48.8)	11 297 (48.4)	31 286 (48.7)	8839 (48.6)
Male	30 225 (51.2)	12 068 (51.7)	32 931 (51.3)	9362 (51.4)
5-min Apgar score				
0-6	271 (0.5)	137 (0.6)	294 (0.5)	114 (0.6)
7-10	58 782 (99.5)	23 228 (99.4)	63 923 (99.5)	18 088 (99.4)
Major congenital anomaly				
No	57 796 (97.9)	22 767 (97.4)	62 816 (97.8)	17 747 (97.5)
Yes	1257 (2.1)	598 (2.6)	1401 (2.2)	454 (2.5)
Feeding method				
Infant formula	14 867 (25.2)	6899 (29.5)	16 677 (26.0)	5088 (28.0)
Mixed feeding	21 935 (37.1)	8883 (38.0)	23 751 (37.0)	7068 (38.8)
Breastfeeding	22 251 (37.7)	7583 (32.5)	23 789 (37.0)	6045 (33.2)
Nursery attendance				
No	55 306 (93.7)	21 592 (92.4)	60 013 (93.5)	16 885 (92.8)
Yes	3747 (6.4)	1773 (7.6)	4204 (6.6)	1316 (7.2)
Marital status				
Married	58 246 (98.6)	22 561 (96.6)	63 076 (98.2)	17 731 (97.4)
Unmarried	807 (1.4)	804 (3.4)	1141 (1.8)	470 (2.6)
Employed				
No	28 455 (48.2)	10 818 (46.3)	31 133 (48.5)	8140 (44.7)
Yes	30 598 (51.8)	12 547 (53.7)	33 085 (51.5)	10 061 (55.3)
Child living with parent(s)				
No	118 (0.2)	80 (0.3)	129 (0.2)	69 (0.4)
Yes	58 935 (99.8)	23 285 (99.7)	64 088 (99.8)	18 132 (99.6)
Child living with sibling(s)				
No	26 513 (44.9)	11 181 (47.9)	29 277 (45.6)	8417 (46.2)
Yes	32 540 (55.1)	12 184 (52.2)	34 940 (54.4)	9784 (53.8)
Child living with mother’s parent(s)				
No	54 194 (91.8)	20 882 (89.4)	58 585 (91.2)	16 491 (90.6)
Yes	4859 (8.2)	2483 (10.6)	5632 (8.8)	1710 (9.4)
Child living with father’s parent(s)				
No	51 990 (88.0)	20 240 (86.6)	56 525 (88.0)	15 705 (86.3)
Yes	7063 (12.0)	3125 (13.4)	7693 (12.0)	2496 (13.7)
Pet ownership type				
None	47 170 (79.9)	17 831 (76.3)	50 858 (79.2)	14 143 (77.7)
Dog(s) only	7453 (12.6)	3278 (14.0)	8371 (13.0)	2360 (13.0)
Cat(s) only	3220 (5.5)	1620 (6.9)	3600 (5.6)	1240 (6.8)
Both	1210 (2.1)	636 (2.7)	1388 (2.2)	458 (2.5)
Stressful event				
No	34 412 (58.3)	11 388 (48.7)	37 452 (58.3)	8348 (45.9)
Yes	24 641 (41.7)	11 977 (51.3)	26 765 (41.7)	9853 (54.1)
Child neurodevelopmental delay				
No	42 657 (72.2)	16 741 (71.6)	46 730 (72.8)	12 668 (69.6)
Yes	16 396 (27.8)	6624 (28.4)	17 487 (27.2)	5533 (30.4)

Abbreviations: BMI, body mass index (calculated as weight in kilograms divided by height in meters squared); PUFA, polyunsaturated fatty acid.

^a^ To convert Japanese yen to US dollars, multiply by 0.0068.

^b^ Quintiles overlap due to multiple imputation.

**Figure 3.**
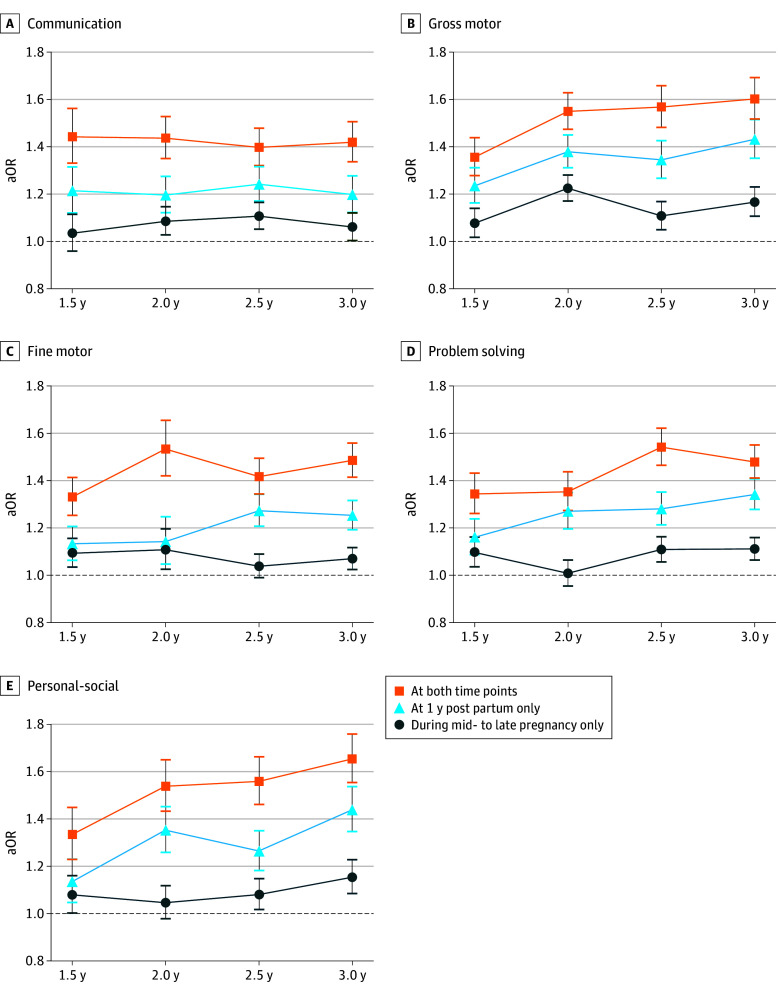
Counterfactual Adjusted Odds Ratios (aORs) for Developmental Delay Graphs illustrate aORs with SEMs (depicted by the whiskers) for each developmental area of the Ages and Stages Questionnaire, Third Edition, across 4 time points, assuming mothers had psychological distress—contrary to fact—during mid- to late pregnancy only, at 1 year post partum only, or at both time points, with not having psychological distress at either time point as the reference. SEMs were estimated using marginal structural modeling with inverse probability of treatment weighting. Weights were calculated using all variables: psychological distress during pregnancy and at 1 year post partum, neurodevelopmental delay at age 0.5 to 1.5 years, baseline potential confounders (defined as *V *in the Statistical Analysis subsection of the Methods section); and post-childbirth time-varying covariates (defined as *L_1_* in the Statistical Analysis subsection of the Methods section).

Table 2 shows the aORs for neurodevelopmental delay measured using the ASQ-3 if mothers had experienced psychological distress during mid- to late pregnancy only, at 1 year post partum only, or at both time points, compared with not having psychological distress at either time point. The aORs for during pregnancy only, at 1 year only, and at both time points were 1.08 (95% CI, 1.03-1.13), 1.25 (95% CI, 1.18-1.33), and 1.38 (95% CI, 1.30-1.46), respectively. The aOR for the multiplicative interaction between the 2 time points was 1.02 (95% CI, 0.93-1.11). Figure 3 shows the aORs across the 4 time points for delays in each of the 5 areas of the ASQ-3 compared with not having psychological distress at either time point. The aORs for psychological distress during pregnancy only were relatively smaller (eg, personal-social skills at age 3 years: aOR, 1.15; 95% CI, 1.02-1.30). In contrast, the aORs for the psychological distress at 1 year post partum only were relatively larger (eg, personal-social skills at age 3 years: aOR, 1.44; 95% CI, 1.26-1.64). At all time points and in all domains, aORs for psychological distress at 1 year post partum only were higher than those for psychological distress during mid- to late pregnancy only. The aORs for psychological distress at both time points were higher as well (eg, personal-social skills at age 3 years: aOR, 1.65; 95% CI, 1.46-1.87). The aORs for the multiplicative interaction ranged from 0.92 to 1.21, all of which had 95% CIs crossing 1.00.

**Table 2.** Counterfactual Odds Ratios for Neurodevelopmental Delay^a^

Model^c^	Psychological distress^b^			
	None (n = 51 348)	During mid- to late pregnancy only (n = 12 869)	At 1 y post partum only (n = 7705)	At both time points (n = 10 496)
Unweighted	1 [Reference]	1.09 (1.04-1.14)	1.36 (1.29-1.43)	1.49 (1.42-1.56)
IPTW				
Crude	1 [Reference]	1.11 (1.06-1.16)	1.30 (1.23-1.37)	1.44 (1.38-1.51)
Partially adjusted	1 [Reference]	1.09 (1.04-1.14)	1.27 (1.20-1.35)	1.41 (1.33-1.49)
Fully adjusted	1 [Reference]	1.08 (1.03-1.13)	1.25 (1.18-1.32)	1.37 (1.29-1.46)

Abbreviation: IPTW, inverse probability of treatment weighting.

^a^ Odds ratios were estimated using marginal structural modeling with IPTW. Neurodevelopmental delay was defined as a score less than the cutoff^40^ in any of the 5 developmental areas at any of the 4 measurement time points (ie, ages 1.5, 2.0, 2.5, and 3.0 years). Of 82 418 toddlers, 23 007 met this criterion.

^b^ Defined as a Kessler Psychological Distress Scale^36^ score of 5 or greater.^37,38^

^c^ For descriptions of the models, see the Statistical Analysis subsection in the Methods section.

Sensitivity analyses using different cutoffs yielded similar or slightly stronger results compared with those derived from the main analysis (eTables 1-2 in Supplement 1). The E-values for aORs for psychological distress during pregnancy only (1.08; 95% CI, 1.03-1.13), at 1 year post partum only (1.25; 95% CI, 1.18-1.33), and at both time points (1.38; 95% CI, 1.30-1.46) in the main analysis and for the highest aOR (1.65; 95% CI, 1.46-1.87 in the secondary analysis) were 1.30 (95% CI, 1.16-1.41), 1.63 (95% CI, 1.51-1.74), 1.82 (95% CI, 1.70-1.93), and 2.63 (95% CI, 2.24-3.05), respectively. Balance diagnoses did not reveal any particular issues (eTables 3-5 and eFigures 1-9 in Supplement 1).

## Discussion

This study examined the complex interplay between maternal psychological distress during mid- to late pregnancy and at 1 year post partum and delayed neurodevelopment in children aged 1.5 to 3.0 years. We used a marginal structural model with inverse probability weighting to accurately estimate the effect sizes for exposures that are time-varying and bidirectionally associated with outcomes. The results showed a stronger estimated risk for psychological distress at 1 year post partum than during mid- to late pregnancy. This tendency was also true for each risk across all 5 developmental areas and all 4 time points. Furthermore, the results were additive in that no multiplicative interaction was found.

Our finding that maternal psychological distress—whether during pregnancy or the postpartum period—had an estimated higher risk of neurodevelopmental delay is broadly consistent with previous findings.^4,5,6,63,64,65^ However, the weaker risk estimate for psychological distress during pregnancy is less consistent with some studies.^28,30,31^ This disparity may stem from differences in model adjustment: earlier studies tended not to adjust for necessary potential confounders, such as psychological distress during pregnancy,^22,66^ gestational age, birth weight,^17,18^ breastfeeding,^14,15^ and child development,^19^ or inadequately adjusted for unnecessary potential intermediate variables such as postpartum psychological distress,^29^ gestational age, and birth weight,^17,18^ limiting causal interpretation. In contrast, our use of a marginal structural model with inverse probability weighting^32,33,42,43^ enabled proper adjustment by treating time-varying bidirectional associations, such as reverse causality, via incorporation of variables that are both confounders and intermediating variables into a single model,^45^ thereby allowing us to independently estimate the effect sizes at each time point. Thus, while the research topics are similar, the estimands are different, and our results suggest a potential causal effect, whereas previous findings are largely associational.

The results of the present study suggest that the prevention, detection, and alleviation of psychological distress not only during the postpartum period but also during pregnancy may play an important role in reducing the risk of developmental delays in toddlers. Because the background mechanisms involved in developmental delay are assumed to differ between pregnancy and the postpartum period,^5,6^ the impact may involve qualitative differences rather than mere differences in magnitude. However, controlling for psychological distress during this period is not straightforward. The perinatal period is known as a vulnerable period for women’s mental health,^67,68^ and in fact, stress during pregnancy occurs due to rapid physical and environmental changes.^69^ There is evidence that postpartum selective serotonin reuptake inhibitor use^70^ and counseling during pregnancy^63^ are effective, but from a public health perspective, it is important to prepare thoroughly before pregnancy.^71^ In addition to avoiding unwanted pregnancies,^72^ it is important to take various approaches that lead to improved mental health, including by enhancing social support^45,54,73^ and adopting a healthier lifestyle in terms of diet,^51,74,75,76^ sleep,^77^ and physical activity.^78^

Several assumptions are necessary for the marginal structure model.^32,42^ Conditional exchangeability was assumed to be satisfied through careful and comprehensive selection of confounders. Selection bias was minimized through efficient imputation using the relatively high correlation with ASQ-3. Regarding unmeasured confounding, sensitivity analysis using E-values indicated that relatively strong unmeasured confounding would be required to cancel out the observed results. Positivity is likely satisfied based on descriptive statistics (Table 1). Correct model specification was likely met as the SW mean was 1—a necessary condition (eTable 3 in Supplement 1). Regarding consistency, psychological distress is inherently considered continuous, and its cause varies among individuals, potentially leading to different outcome. Therefore, we classified psychological distress as 0 or 1 using validated cutoff values and created a summary indicator of any neurodevelopmental delay to address this diversity, assuming that it was satisfied within a certain range.

### Strengths and Limitations

This study has the following strengths. First, we analyzed data from an ongoing nationwide birth cohort (exceeding 82 000 mother-child pairs) from both urban and rural areas in Japan, and thus, the sample can be considered representative of Japanese mothers. Second, exposure was measured once before and once after delivery, and the outcome was measured every 6 months from 1.5 to 3.0 years after delivery, for a total of 4 times. This allowed us to obtain rich results. Third, we used a marginal structural model to handle time-varying exposures. Hence, this is likely to result in more reasonable estimates than before.

This study also has some limitations. First, we measured neurodevelopmental delay using the ASQ-3. Although both the ASQ-3 and K6 are well-validated questionnaires, they are neither diagnostic nor objective. Additionally, while the ASQ-3 has multiple domains and detailed age-specific settings, its screening performance may not be consistent across all age groups. This raises the possibility of misclassification, particularly in the outcome measurement. Furthermore, mothers with psychological distress may view their child’s neurodevelopment pessimistically. Taken together, these might result in inaccurate estimates and potential overestimation. Second, maternal psychological distress is likely a cause of nonresponse and dropout, possibly resulting in selection bias. Third, efforts were made to avoid unmeasured confounders by using many variables, but the presence of confounders is likely and may have resulted in overestimation. Furthermore, the generalizability of the current findings must be examined in other settings outside Japan because psychological distress and neurodevelopmental delay are likely to be influenced by various factors.

## Conclusions

In this cohort study of 82 418 mother-child pairs, maternal psychological distress had an estimated higher risk of neurodevelopmental delay in toddlers both during mid- to late pregnancy and at 1 year post partum, with a stronger risk estimate observed in the latter period. These findings highlight the importance of maintaining maternal mental health from pregnancy through 1 year post partum.

## Notes

Supplement 1. **eTable 1.** Counterfactual Odds Ratios for Neurodevelopmental Delay **eTable 2.** Counterfactual Odds Ratios for Continuous Severity Score of Neurodevelopmental Delay **eTable 3.** Distribution of the Estimated Weights in the Main Analysis **eTable 4.** Distribution of the Estimated Weights in the Sensitivity Analysis (Using a Kessler Psychological Distress Scale Cutoff Value of 13) **eTable 5.** Distributions of the Estimated Weights in the Sensitivity Analysis (Using a Continuous Severity Score of Neurodevelopmental Delay) **eFigure 1.** Distribution Balance for PsyD_1_ and PsyD_2_ and Covariate Balance Before and After Weighting for PsyD_1_ and PsyD_2_ in the Fully Adjusted Model in the Main Analysis **eFigure 2.** Distribution Balance for PsyD_1_ and PsyD_2_ and Covariate Balance Before and After Weighting for PsyD_1_ and PsyD_2_ in the Partially Adjusted Model in the Main Analysis **eFigure 3.** Distribution Balance for PsyD_2_ and Covariate Balance Before and After Weighting for PsyD_2_ in the Crude Model in the Main Analysis **eFigure 4.** Distribution Balance for PsyD_1_ and PsyD_2_ and Covariate Balance Before and After Weighting for PsyD_1_ and PsyD_2_ in the Fully Adjusted Model in the Sensitivity Analysis (Using a Kessler Psychological Distress Scale Cutoff Value of 13) **eFigure 5.** Distribution Balance for PsyD_1_ and PsyD_2_ and Covariate Balance Before and After Weighting for PsyD_1_ and PsyD_2_ in the Partially Adjusted Model in the Sensitivity Analysis (Using a Kessler Psychological Distress Scale Cutoff Value of 13) **eFigure 6.** Distribution Balance for PsyD_2_ and Covariate Balance Before and After Weighting for PsyD_2_ in the Crude Model in the Sensitivity Analysis (Using a Kessler Psychological Distress Scale Cutoff Value of 13) **eFigure 7.** Distribution Balance for PsyD_1_ and PsyD_2_ and Covariate Balance Before and After Weighting for PsyD_1_ and PsyD_2_ in the Fully Adjusted Model in the Sensitivity Analysis (Using a Continuous Severity Score of Neurodevelopmental Delay) **eFigure 8.** Distribution Balance for PsyD_1_ and PsyD_2_ and Covariate Balance Before and After Weighting for PsyD_1_ and PsyD_2_ in the Partially Adjusted Model in the Sensitivity Analysis (Using a Continuous Severity Score of Neurodevelopmental Delay) **eFigure 9.** Distribution Balance for PsyD_2_ and Covariate Balance Before and After Weighting for PsyD_2_ in the Crude Model in the Sensitivity Analysis (Using a Continuous Severity Score of Neurodevelopmental Delay)

Supplement 2.
Nonauthor Collaborators

Supplement 3.
Data Sharing Statement
