# Supplementary material for: Maternal Psychological Distress Before and After Childbirth and Neurodevelopmental Delay in Toddlers
Source: JAMA Netw Open. 2025 Oct 31;8(10):e2540907. doi: 10.1001/jamanetworkopen.2025.40907 (PMC12579353; doi:10.1001/jamanetworkopen.2025.40907)
Supplement: Supplement 1. — eTable 1. Counterfactual Odds Ratios for Neurodevelopmental Delay eTable 2. Counterfactual Odds Ratios for Continuous Severity Score of Neurodevelopmental Delay eTable 3. Distribution of the Estimated Weights in the Main Analysis eTable 4. Distribution of the Estimated Weights in the Sensitivity Analysis (Using a Kessler Psychological Distress Scale Cutoff Value of 13) eTable 5. Distributions of the Estimated Weights in the Sensitivity Analysis (Using a Continuous Severity Score of Neurodevelopmental Delay) eFigure 1. Distribution Balance for PsyD1 and PsyD2 and Covariate Balance Before and After Weighting for PsyD1 and PsyD2 in the Fully Adjusted Model in the Main Analysis eFigure 2. Distribution Balance for PsyD1 and PsyD2 and Covariate Balance Before and After Weighting for PsyD1 and PsyD2 in the Partially Adjusted Model in the Main Analysis eFigure 3. Distribution Balance for PsyD2 and Covariate Balance Before and After Weighting for PsyD2 in the Crude Model in the Main Analysis eFigure 4. Distribution Balance for PsyD1 and PsyD2 and Covariate Balance Before and After Weighting for PsyD1 and PsyD2 in the Fully Adjusted Model in the Sensitivity Analysis (Using a Kessler Psychological Distress Scale Cutoff Value of 13) eFigure 5. Distribution Balance for PsyD1 and PsyD2 and Covariate Balance Before and After Weighting for PsyD1 and PsyD2 in the Partially Adjusted Model in the Sensitivity Analysis (Using a Kessler Psychological Distress Scale Cutoff Value of 13) eFigure 6. Distribution Balance for PsyD2 and Covariate Balance Before and After Weighting for PsyD2 in the Crude Model in the Sensitivity Analysis (Using a Kessler Psychological Distress Scale Cutoff Value of 13) eFigure 7. Distribution Balance for PsyD1 and PsyD2 and Covariate Balance Before and After Weighting for PsyD1 and PsyD2 in the Fully Adjusted Model in the Sensitivity Analysis (Using a Continuous Severity Score of Neurodevelopmental Delay) eFigure 8. Distribution Balance for PsyD1 and PsyD2 and C [file jamanetwopen-e2540907-s001.pdf]

Supplemental Online Content

Matsumura K, Tanaka T, Kuroda M, Tsuchida A, Hatakeyama T, Kasamatsu H, Inadera H. Maternal psychological distress before and after childbirth and neurodevelopmental delay in toddlers. *JAMA Netw Open*. 2025;8(10):e2540907. doi:10.1001/jamanetworkopen.2025.40907

eTable 1. Counterfactual Odds Ratios for Neurodevelopmental Delay

eTable 2. Counterfactual Odds Ratios for Continuous Severity Score of Neurodevelopmental Delay

eTable 3. Distribution of the Estimated Weights in the Main Analysis

eTable 4. Distribution of the Estimated Weights in the Sensitivity Analysis (Using a Kessler Psychological Distress Scale Cutoff Value of 13)

eTable 5. Distributions of the Estimated Weights in the Sensitivity Analysis (Using a Continuous Severity Score of Neurodevelopmental Delay)

eFigure 1. Distribution Balance for $PsyD_1$ and $PsyD_2$ and Covariate Balance Before and After Weighting for $PsyD_1$ and $PsyD_2$ in the Fully Adjusted Model in the Main Analysis

eFigure 2. Distribution Balance for $PsyD_1$ and $PsyD_2$ and Covariate Balance Before and After Weighting for $PsyD_1$ and $PsyD_2$ in the Partially Adjusted Model in the Main Analysis

eFigure 3. Distribution Balance for $PsyD_2$ and Covariate Balance Before and After Weighting for $PsyD_2$ in the Crude Model in the Main Analysis

eFigure 4. Distribution Balance for $PsyD_1$ and $PsyD_2$ and Covariate Balance Before and After Weighting for $PsyD_1$ and $PsyD_2$ in the Fully Adjusted Model in the Sensitivity Analysis (Using a Kessler Psychological Distress Scale Cutoff Value of 13)

eFigure 5. Distribution Balance for $PsyD_1$ and $PsyD_2$ and Covariate Balance Before and After Weighting for $PsyD_1$ and $PsyD_2$ in the Partially Adjusted Model in the Sensitivity Analysis (Using a Kessler Psychological Distress Scale Cutoff Value of 13)

© 2025 Matsumura K et al. *JAMA Network Open*.

eFigure 6. Distribution Balance for $PsyD_2$ and Covariate Balance Before and After Weighting for $PsyD_2$ in the Crude Model in the Sensitivity Analysis (Using a Kessler Psychological Distress Scale Cutoff Value of 13)

eFigure 7. Distribution Balance for $PsyD_1$ and $PsyD_2$ and Covariate Balance Before and After Weighting for $PsyD_1$ and $PsyD_2$ in the Fully Adjusted Model in the Sensitivity Analysis (Using a Continuous Severity Score of Neurodevelopmental Delay)

eFigure 8. Distribution Balance for $PsyD_1$ and $PsyD_2$ and Covariate Balance Before and After Weighting for $PsyD_1$ and $PsyD_2$ in the Partially Adjusted Model in the Sensitivity Analysis (Using a Continuous Severity Score of Neurodevelopmental Delay)

eFigure 9. Distribution Balance for $PsyD_2$ and Covariate Balance Before and After Weighting for $PsyD_2$ in the Crude Model in the Sensitivity Analysis (Using a Continuous Severity Score of Neurodevelopmental Delay)

This supplemental material has been provided by the authors to give readers additional information about their work.

© 2025 Matsumura K et al. *JAMA Network Open*.

eTable 1. Counterfactual Odds Ratios[a] for Neurodevelopmental Delay[b]

Model	Psychological distress[c]			
	None (n = 78,333)	During mid/late pregnancy only (n = 1,946)	At 1 year postpartum only (n = 1,568)	At both time points (n = 571)
Unweighted[d]	1.00 (Reference)	1.16 (1.04–1.29)	1.44 (1.30–1.61)	1.71 (1.42–2.06)
IPTW				
Crude[e]	1.00 (Reference)	1.17 (1.05–1.30)	1.42 (1.28–1.58)	1.69 (1.40–2.03)
Partially adjusted[f]	1.00 (Reference)	1.18 (1.05–1.33)	1.38 (1.23–1.56)	1.75 (1.40–2.19)
Fully adjusted[g]	1.00 (Reference)	1.18 (1.04–1.34)	1.34 (1.19–1.52)	1.74 (1.37–2.21)

[a] Estimated using marginal structural modeling with inverse probability of treatment weightings (IPTW).
[b] Defined as a score below the cut-off[40] in any of the five developmental areas at any of the four measurement time points (i.e., 1.5, 2.0, 2.5, and 3.0 years of age) (n = 23,007).
[c] Defined as a Kessler Psychological Distress Scale[36] score of ≥13[37,38].
[d] Model using weights fixed at 1.
[e] Model using weights calculated with minimum variables (based only on $PsyD_1$, $PsyD_2$, and NDD_1; $\boldsymbol{V}$ and $\boldsymbol{L_1}$ not used, see text)
[f] Model using weights calculated with limited key variables: $PsyD_1$, $PsyD_2$, and NDD_1. $\boldsymbol{V}$: mother's age during pregnancy; pre-pregnancy body mass index (BMI); highest education level[41], annual household income; history of major psychiatric disorders such as depression, anxiety, and schizophrenia; emotional social support[48]; and stressful event. $\boldsymbol{L_1}$: child sex; stressful event; and low birth weight.
[g] Model using weights calculated with all variables: $PsyD_1$, $PsyD_2$, and NDD_1. $\boldsymbol{V}$: mother's age during pregnancy; pre-pregnancy BMI; parity; highest education level[41], annual household income; marital status; physical activity[42,43]; alcohol intake; smoking history; passive smoking; employment status; history of major psychiatric disorders such as depression, anxiety, and schizophrenia; autistic trait assessed using the Autism-Spectrum Quotient (AQ-J-10[44]); folic acid intake[45] and energy-adjusted omega-3 polyunsaturated fatty acid intake[46,47], both assessed using the Food Frequency Questionnaire[48]; emotional social support[49]; negative feelings upon learning of pregnancy; intimate partner violence[50]; living with the mother's parent(s); living with the partner's parent(s); pet ownership[51]; stressful event; and area of residence (one of the 15 regional centers where participants were recruited). $\boldsymbol{L_1}$: caesarean section; preterm birth; low birth weight; child sex; 5-min Apgar score[52]; major congenital anomaly[53]; feeding method[54]; nursery attendance; marital status; employment status; child living with the mother or father; child living with siblings; child living with the mother's parent(s); child living with the partner's parent(s); pet ownership; and stressful event.

© 2025 Matsumura K et al. *JAMA Network Open*.

eTable 2. Counterfactual Odds Ratios[a] for Continuous Severity Score of Neurodevelopmental Delay[b]

Model	Psychological distress[c]			
	None (n = 51,348)	During mid/late pregnancy only (n = 12,869)	At 1 year postpartum only (n = 7,705)	At both time points (n=10,496)
Unweighted[d]	1.00 (Reference)	1.10 (1.07–1.14)	1.35 (1.30–1.40)	1.56 (1.51–1.62)
IPTW				
Crude[e]	1.00 (Reference)	1.12 (1.07–1.17)	1.28 (1.22–1.35)	1.51 (1.44–1.58)
Partially adjusted[f]	1.00 (Reference)	1.09 (1.05–1.15)	1.25 (1.19–1.32)	1.46 (1.39–1.54)
Fully adjusted[g]	1.00 (Reference)	1.08 (1.03–1.13)	1.23 (1.17–1.30)	1.42 (1.35–1.50)

[a] Estimated using marginal structural modeling with inverse probability of treatment weightings (IPTW).
[a] Ddefined as 0 (0 domains below cut-off[40], n = 59,411), 1 (1 domain, n = 10,381), 2 (2–4 domains, n = 8,235), and 3 (≥5 domains, n = 4,391), based on score in five developmental areas across four measurement time points (i.e., 1.5, 2.0, 2.5, and 3.0 years of age).
[b] Defined as a Kessler Psychological Distress Scale[36] score of ≥5[37,38].
[c] Model using weights fixed at 1.
[d] Model using weights calculated with minimum variates (based only on $PsyD_1$, $PsyD_2$, and NDD_1; $\boldsymbol{V}$ and $\boldsymbol{L}_1$ not used, see text). NDD_1 defined as 0 (0 domains below cut-off, n = 59,398), 1 (1 domain, n = 13,186), 2 (2–3 domains, n = 7,698), and 3 (≥4 domains, n = 2,136).
[e] Model using weights calculated with limited key variables: $PsyD_1$, $PsyD_2$, and NDD_1. $\boldsymbol{V}$: mother's age during pregnancy; pre-pregnancy body mass index (BMI); highest education level[41], annual household income; history of major psychiatric disorders such as depression, anxiety, and schizophrenia; emotional social support[48]; and stressful event. $\boldsymbol{L}_1$: child sex; stressful event; and low birth weight.
[f] Model using weights calculated with all variables: $PsyD_1$, $PsyD_2$, and NDD_1. $\boldsymbol{V}$: mother's age during pregnancy; pre-pregnancy BMI; parity; highest education level[41], annual household income; marital status; physical activity[42,43]; alcohol intake; smoking history; passive smoking; employment status; history of major psychiatric disorders such as depression, anxiety, and schizophrenia; autistic trait assessed using the Autism-Spectrum Quotient (AQ-J-10[44]); folic acid intake[45] and energy-adjusted omega-3 polyunsaturated fatty acid intake[46,47], both assessed using the Food Frequency Questionnaire[48]; emotional social support[49]; negative feelings upon learning of pregnancy; intimate partner violence[50]; living with the mother's parent(s); living with the partner's parent(s); pet ownership[51]; stressful event; and area of residence (one of the 15 regional centers where participants were recruited). $\boldsymbol{L}_1$: caesarean section; preterm birth; low birth weight; child sex; 5-min Apgar score[52]; major congenital anomaly[53]; feeding method[54]; nursery attendance; marital status; employment status; child living with the mother or father; child living with siblings; child living with the mother's parent(s); child living with the partner's parent(s); pet ownership; and stressful event.

© 2025 Matsumura K et al. *JAMA Network Open*.

eTable 3. Distribution of the Estimated Weights in the Main Analysis

Weight type	Mean	SD	Median	Percentile			
				1st	25th	75th	99th
**Fully adjusted model**							
$PsyD_1$							
Stabilized weight	0.999	0.368	0.913	0.387	0.827	1.078	2.469
Unstabilized weights	1.999	1.435	1.391	1.103	1.218	2.057	8.067
$PsyD_2$							
Stabilized weight	1.000	0.201	0.979	0.535	0.938	1.041	1.777
Unstabilized weights	1.994	2.087	1.193	1.058	1.117	1.790	11.592
Final (=SW)							
Stabilized weight	1.000	0.458	0.899	0.282	0.788	1.093	2.808
Unstabilized weights	4.008	4.348	1.769	1.180	1.384	5.448	19.563
**Partially adjusted model**							
$PsyD_1$							
Stabilized weight	1.000	0.303	0.941	0.453	0.838	1.079	2.218
Unstabilized weights	2.002	1.328	1.425	1.127	1.246	2.113	7.378
$PsyD_2$							
Stabilized weight	1.000	0.163	0.984	0.601	0.946	1.039	1.642
Unstabilized weights	1.998	2.026	1.191	1.073	1.123	1.834	11.191
Final (=SW)							
Stabilized weight	1.000	0.365	0.928	0.352	0.805	1.096	2.537
Unstabilized weights	4.008	4.022	1.804	1.220	1.426	5.619	18.062
**Crude model**							
$PsyD_1$							
Stabilized weight	1.000	0.000	1.000	1.000	1.000	1.000	1.000
Unstabilized weights	2.000	0.961	1.396	1.396	1.396	3.527	3.527
$PsyD_2$							
Stabilized weight	1.000	0.028	0.994	0.905	0.994	1.016	1.056
Unstabilized weights	2.000	1.866	1.169	1.143	1.143	1.917	7.988
Final (=SW)							
Stabilized weight	1.000	0.028	0.994	0.905	0.994	1.016	1.056
Unstabilized weights	4.000	3.258	1.631	1.595	1.595	6.761	11.148

© 2025 Matsumura K et al. *JAMA Network Open*.

eTable 4. Distribution of the Estimated Weights in the Sensitivity Analysis (Using a Kessler Psychological Distress Scale Cutoff Value of 13)

Weight type	Mean	SD	Median	Percentile			
				1st	25th	75th	99th
**Fully adjusted model**							
$PsyD_1$							
Stabilized weight	0.999	0.368	0.913	0.387	0.827	1.078	2.469
Unstabilized weights	1.999	1.435	1.391	1.103	1.218	2.057	8.067
$PsyD_2$							
Stabilized weight	1.000	0.201	0.979	0.535	0.938	1.041	1.777
Unstabilized weights	1.994	2.087	1.193	1.058	1.117	1.790	11.592
Final (=SW)							
Stabilized weight	1.000	0.458	0.899	0.282	0.788	1.093	2.808
Unstabilized weights	4.008	4.348	1.769	1.180	1.384	5.448	19.563
**Partially adjusted model**							
$PsyD_1$							
Stabilized weight	1.000	0.303	0.941	0.453	0.838	1.079	2.218
Unstabilized weights	2.002	1.328	1.425	1.127	1.246	2.113	7.378
$PsyD_2$							
Stabilized weight	1.000	0.163	0.984	0.601	0.946	1.039	1.642
Unstabilized weights	1.998	2.026	1.191	1.073	1.123	1.834	11.191
Final (=SW)							
Stabilized weight	1.000	0.365	0.928	0.352	0.805	1.096	2.537
Unstabilized weights	4.008	4.022	1.804	1.220	1.426	5.619	18.062
**Crude model**							
$PsyD_1$							
Stabilized weight	1.000	0.000	1.000	1.000	1.000	1.000	1.000
Unstabilized weights	2.000	0.961	1.396	1.396	1.396	3.527	3.527
$PsyD_2$							
Stabilized weight	1.000	0.028	0.994	0.905	0.994	1.016	1.056
Unstabilized weights	2.000	1.866	1.169	1.143	1.143	1.917	7.988
Final (=SW)							
Stabilized weight	1.000	0.028	0.994	0.905	0.994	1.016	1.056
Unstabilized weights	4.000	3.258	1.631	1.595	1.595	6.761	11.148

© 2025 Matsumura K et al. *JAMA Network Open*.

eTable 5. Distributions of the Estimated Weights in the Sensitivity Analysis (Using a Continuous Severity Score of Neurodevelopmental Delay)

Weight type	Mean	SD	Median	Percentile			
				1st	25th	75th	99th
**Fully adjusted model**							
$PsyD_1$							
Stabilized weight	0.999	0.368	0.913	0.387	0.827	1.078	2.469
Unstabilized weights	1.999	1.435	1.391	1.103	1.218	2.057	8.067
$PsyD_2$							
Stabilized weight	1.000	0.201	0.979	0.535	0.938	1.041	1.777
Unstabilized weights	1.994	2.087	1.193	1.058	1.117	1.790	11.592
Final (=SW)							
Stabilized weight	1.000	0.458	0.899	0.282	0.788	1.093	2.808
Unstabilized weights	4.008	4.348	1.769	1.180	1.384	5.448	19.563
**Partially adjusted model**							
$PsyD_1$							
Stabilized weight	1.000	0.303	0.941	0.453	0.838	1.079	2.218
Unstabilized weights	2.002	1.328	1.425	1.127	1.246	2.113	7.378
$PsyD_2$							
Stabilized weight	1.000	0.163	0.984	0.601	0.946	1.039	1.642
Unstabilized weights	1.998	2.026	1.191	1.073	1.123	1.834	11.191
Final (=SW)							
Stabilized weight	1.000	0.365	0.928	0.352	0.805	1.096	2.537
Unstabilized weights	4.008	4.022	1.804	1.220	1.426	5.619	18.062
**Crude model**							
$PsyD_1$							
Stabilized weight	1.000	0.000	1.000	1.000	1.000	1.000	1.000
Unstabilized weights	2.000	0.961	1.396	1.396	1.396	3.527	3.527
$PsyD_2$							
Stabilized weight	1.000	0.028	0.994	0.905	0.994	1.016	1.056
Unstabilized weights	2.000	1.866	1.169	1.143	1.143	1.917	7.988
Final (=SW)							
Stabilized weight	1.000	0.028	0.994	0.905	0.994	1.016	1.056
Unstabilized weights	4.000	3.258	1.631	1.595	1.595	6.761	11.148

© 2025 Matsumura K et al. *JAMA Network Open*.

eFigure 1. Distribution Balance for $PsyD_1$ (Panel A) and $PsyD_2$ (Panel B) and Covariate Balance Before and After Weighting for $PsyD_1$ (Panel C) and $PsyD_2$ (Panel D) in the Fully Adjusted Model in the Main Analysis

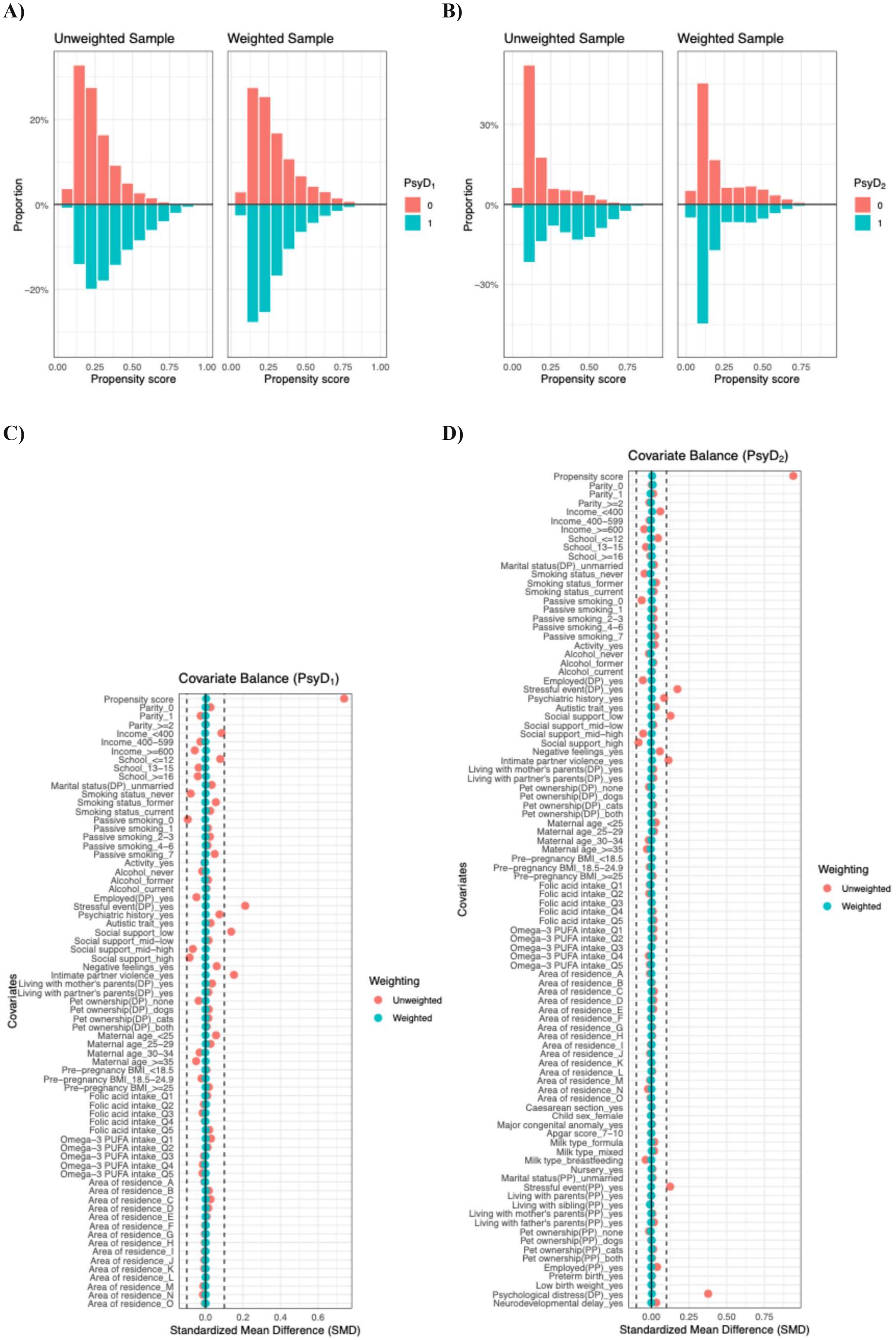

© 2025 Matsumura K et al. *JAMA Network Open*.

eFigure 2. Distribution Balance for $PsyD_1$ (Panel A) and $PsyD_2$ (Panel B) and Covariate Balance Before and After Weighting for $PsyD_1$ (Panel C) and $PsyD_2$ (Panel D) in the Partially Adjusted Model in the Main Analysis

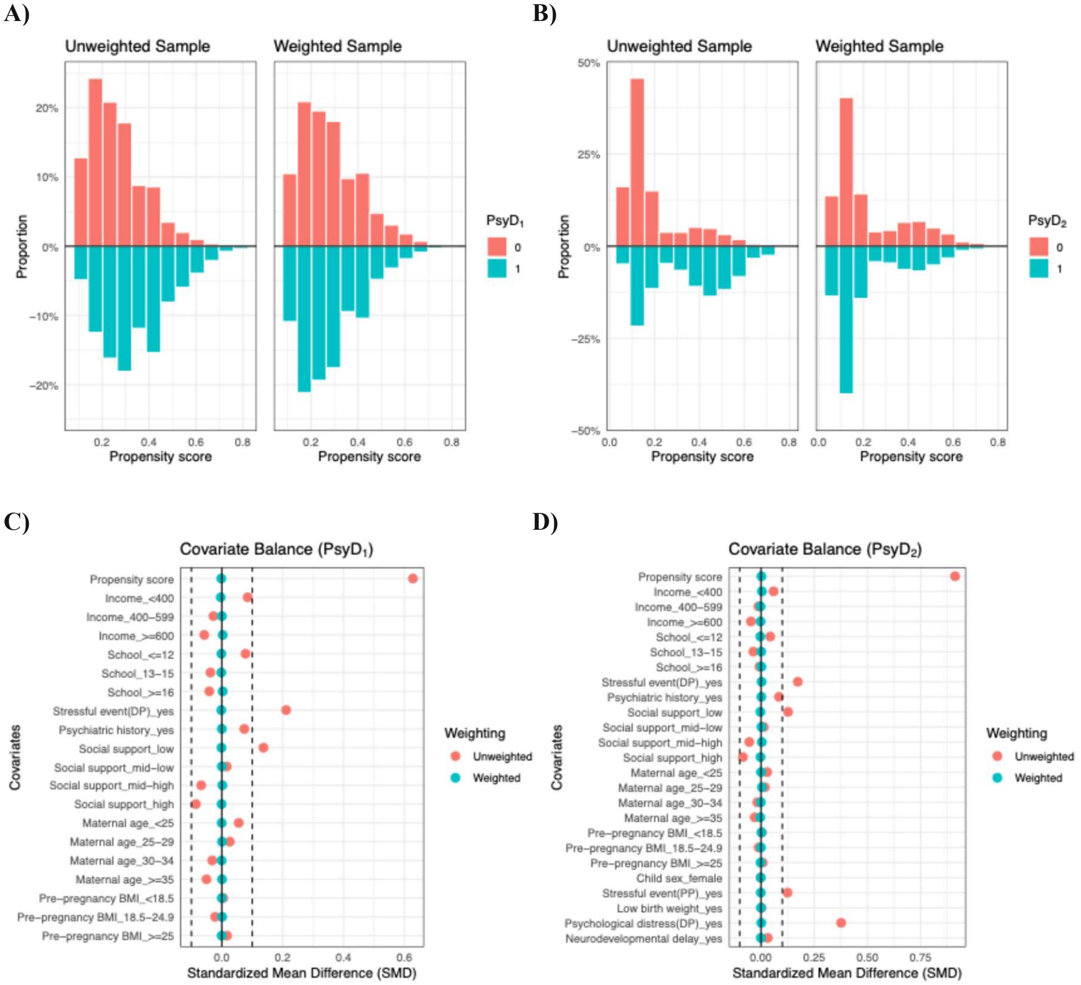

© 2025 Matsumura K et al. *JAMA Network Open*.

eFigure 3. Distribution Balance for $PsyD_2$ (Panel A) and Covariate Balance Before and After Weighting for $PsyD_2$ (Panel B) in the Crude Model in the Main Analysis

A) **B)**

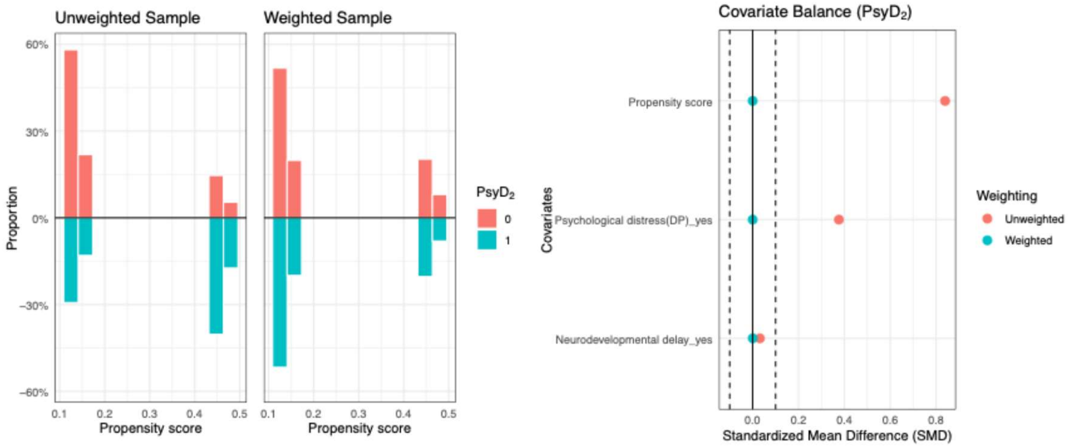

Because this is a crude model, it was not possible to depict the distribution balance and covariate balance for $PsyD_1$.

© 2025 Matsumura K et al. *JAMA Network Open*.

eFigure 4. Distribution Balance for $PsyD_1$ (Panel A) and $PsyD_2$ (Panel B) and Covariate Balance Before and After Weighting for $PsyD_1$ (Panel C) and $PsyD_2$ (Panel D) in the Fully Adjusted Model in the Sensitivity Analysis (Using a Kessler Psychological Distress Scale Cutoff Value of 13)

A)

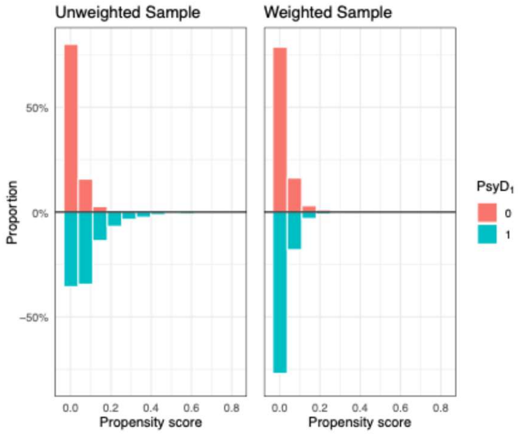

B)

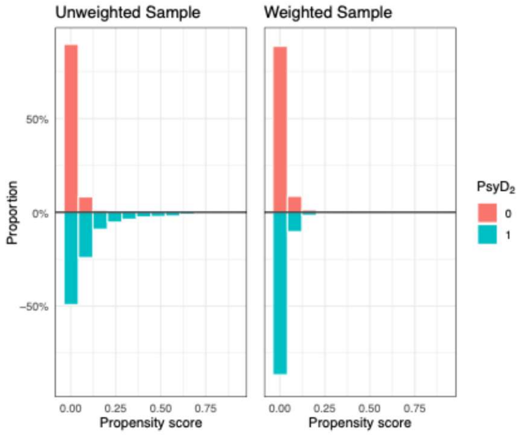

C)

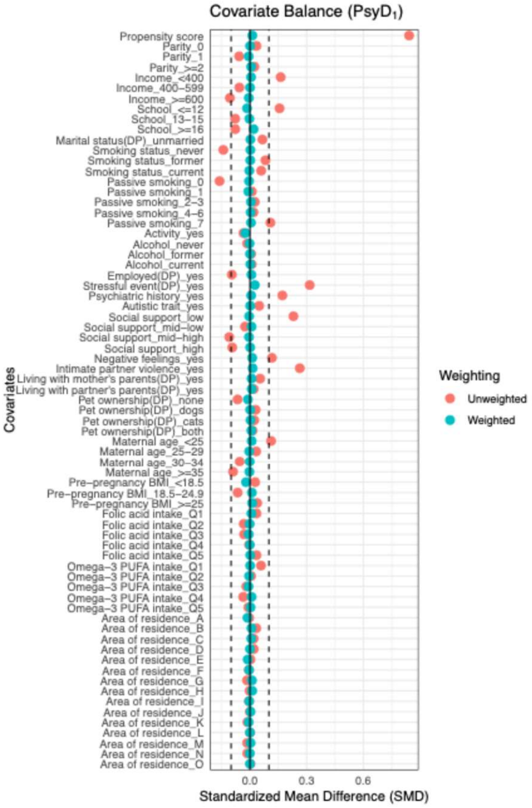

D)

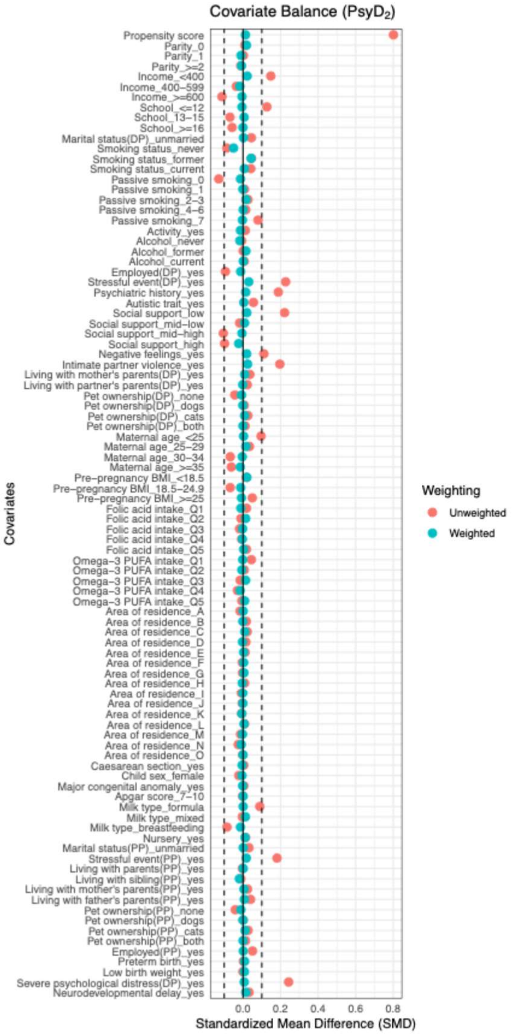

© 2025 Matsumura K et al. *JAMA Network Open*.

eFigure 5. Distribution Balance for $PsyD_1$ (Panel A) and $PsyD_2$ (Panel B) and Covariate Balance Before and After Weighting for $PsyD_1$ (Panel C) and $PsyD_2$ (Panel D) in the Partially Adjusted Model in the Sensitivity Analysis (Using a Kessler Psychological Distress Scale Cutoff Value of 13)

A)

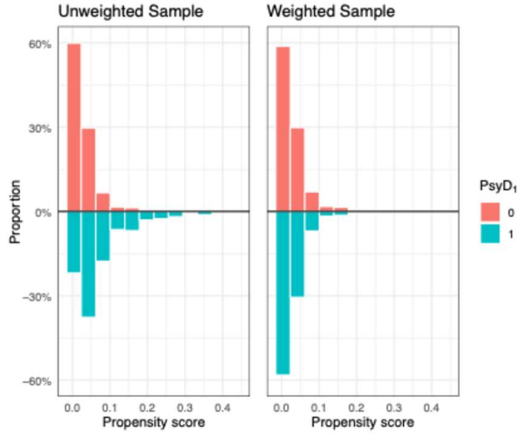

B)

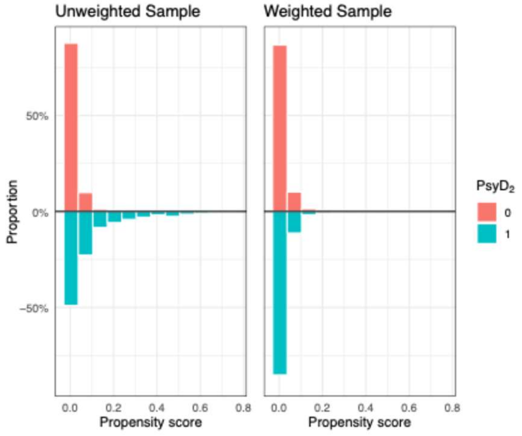

C)

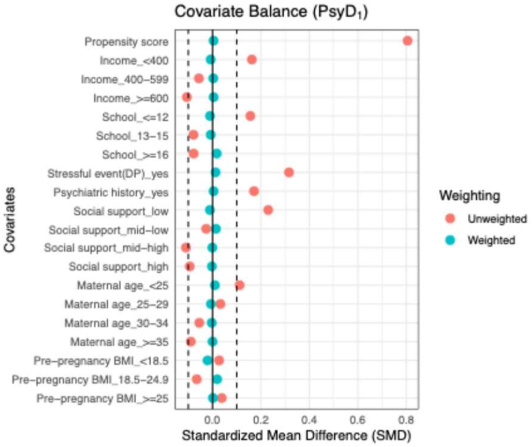

D)

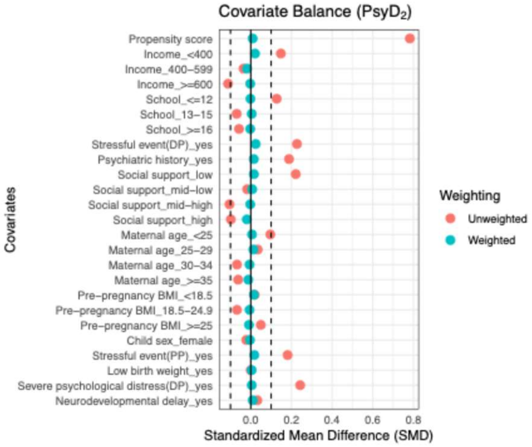

© 2025 Matsumura K et al. *JAMA Network Open*.

eFigure 6. Distribution Balance for $PsyD_2$ (Panel A) and Covariate Balance Before and After Weighting for $PsyD_2$ (Panel B) in the Crude Model in the Sensitivity Analysis (Using a Kessler Psychological Distress Scale Cutoff Value of 13)

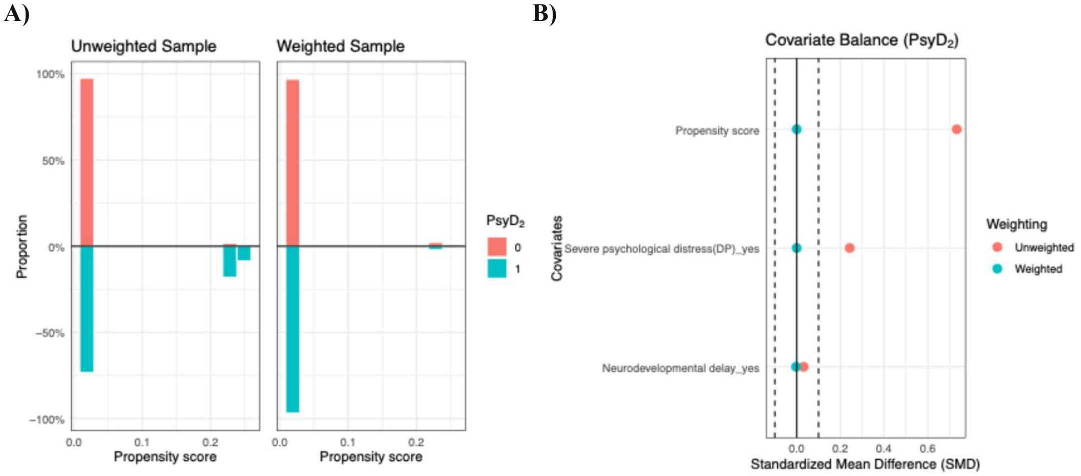

Because this is a crude model, it was not possible to depict the distribution balance and covariate balance for $PsyD_1$.

eFigure 7. Distribution Balance for $PsyD_1$ (Panel A) and $PsyD_2$ (Panel B) and Covariate Balance Before and After Weighting for $PsyD_1$ (Panel C) and $PsyD_2$ (Panel D) in the Fully Adjusted Model in the Sensitivity Analysis (Using a Continuous Severity Score of Neurodevelopmental Delay)

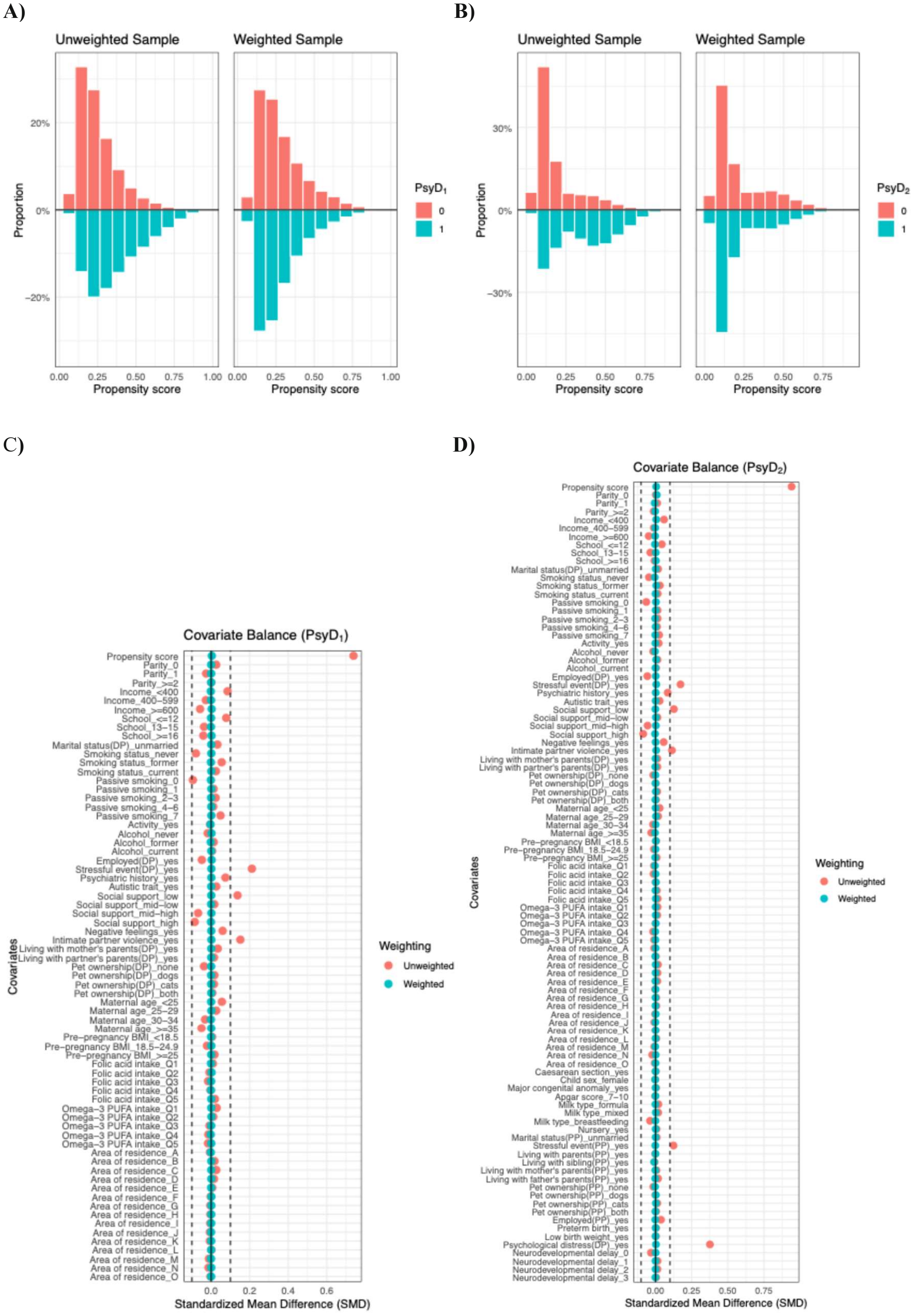

eFigure 8. Distribution Balance for PsyD$_1$ (Panel A) and PsyD$_2$ (Panel B) and Covariate Balance Before and After Weighting for PsyD$_1$ (Panel C) and PsyD$_2$ (Panel D) in the Partially Adjusted Model in the Sensitivity Analysis (Using a Continuous Severity Score of Neurodevelopmental Delay)

A)

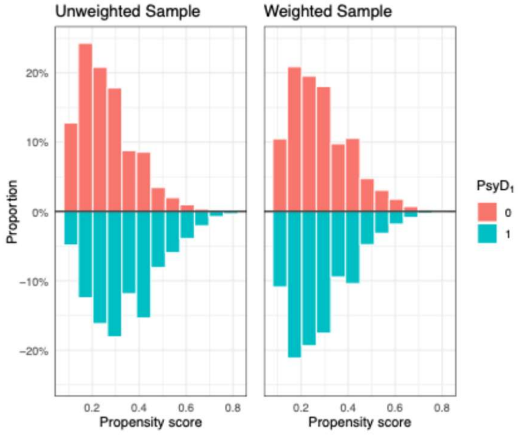

B)

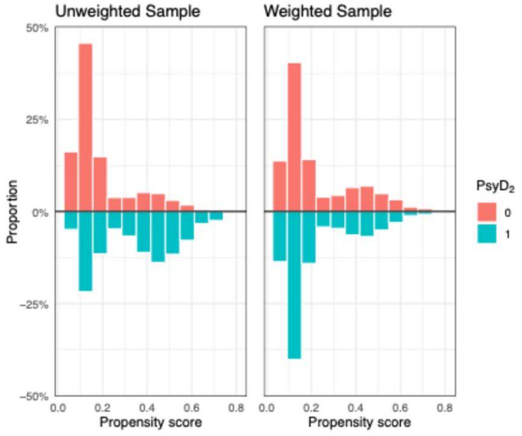

C)

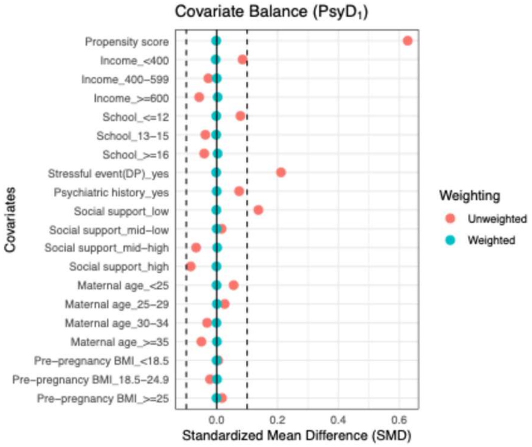

D)

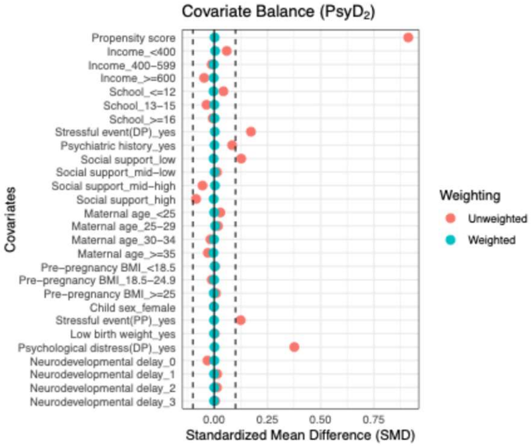

© 2025 Matsumura K et al. *JAMA Network Open*.

eFigure 9. Distribution Balance for PsyD$_2$ (Panel A) and Covariate Balance Before and After Weighting for PsyD$_2$ (Panel B) in the Crude Model in the Sensitivity Analysis (Using a Continuous Severity Score of Neurodevelopmental Delay)

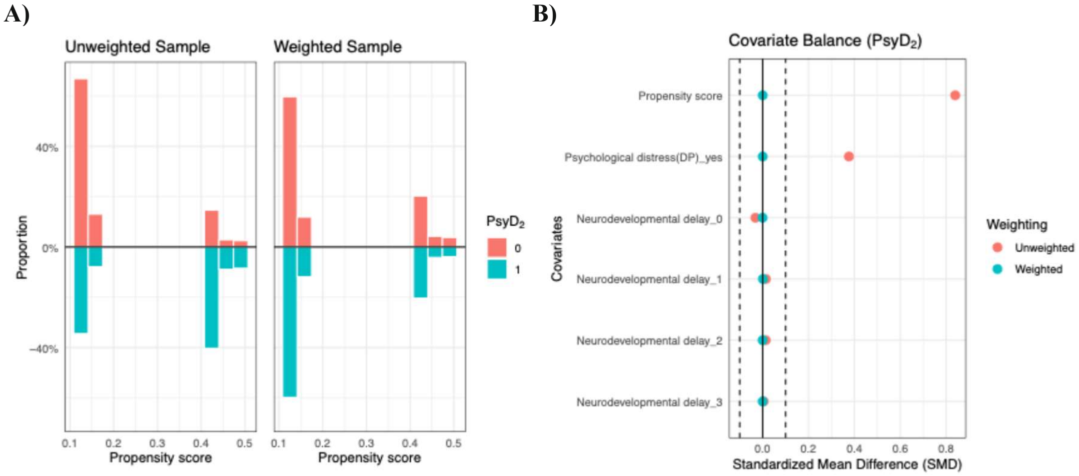

Because this is a crude model, it was not possible to depict the distribution balance and covariate balance for PsyD$_1$.

© 2025 Matsumura K et al. *JAMA Network Open*.
