# Supplementary material for: Maternal Psychological Distress Before and After Childbirth and Neurodevelopmental Delay in Toddlers
Source: JAMA Netw Open. 2025 Oct 31;8(10):e2540907. doi: 10.1001/jamanetworkopen.2025.40907 (PMC12579353; doi:10.1001/jamanetworkopen.2025.40907)
Supplement: Supplement 3. — Data Sharing Statement [file jamanetwopen-e2540907-s003.pdf]

Data Sharing Statement

Matsumura. Maternal Psychological Distress Before and After Childbirth and Neurodevelopmental Delay in Toddlers. *JAMA Netw Open*. Published October 31, 2025. doi:10.1001/jamanetworkopen.2025.40907

Data
Data available: No

Additional Information
Explanation for why data not available: Data are unsuitable for public deposition due to ethical restrictions and the legal framework of Japan. It is prohibited by the Act on the Protection of Personal Information (Act No. 57 of 30 May 2003, amendment 9 September 2015) to publicly deposit the data containing personal information. Ethical Guidelines for Medical and Health Research Involving Human Subjects enforced by the Japanese Ministry of Education, Culture, Sports, Science and Technology and the Ministry of Health, Labour and Welfare also restrict the open sharing of the epidemiologic data. All inquiries about access to data should be sent to: jecs-en@nies.go.jp. The person responsible for handling inquiries sent to this e-mail address is Dr Shoji F. Nakayama, JECS Programme Office, National Institute for Environmental Studies.
